# Isotope Fractionation during Gas Chromatography Can Enhance Mass Spectrometry-Based Measures of ^2^H-Labeling of Small Molecules

**DOI:** 10.3390/metabo10110474

**Published:** 2020-11-20

**Authors:** Daniel P. Downes, Takhar Kasumov, Natalie A. Daurio, Neil B. Wood, Michael J. Previs, Payal R. Sheth, David G. McLaren, Stephen F. Previs

**Affiliations:** 1 Department of Chemistry, Merck & Co., Inc., Kenilworth, NJ 07033, USA; daniel.downes@merck.com (D.P.D.); natalie.daurio@pfizer.com (N.A.D.); payal.sheth@merck.com (P.R.S.); david_mclaren@merck.com (D.G.M.); 2 Department of Pharmaceutical Sciences, Northeast Ohio Medical University, Rootstown, OH 44272, USA; tkasumov@neomed.edu; 3 Department of Molecular Physiology & Biophysics, University of Vermont, Burlington, VT 05405, USA; neil.wood@uvm.edu (N.B.W.); mprevis@uvm.edu (M.J.P.)

**Keywords:** isotope fractionation, gas chromatography-mass spectrometry, stable isotopes, metabolic flux, data integration, Savitzky-Golay

## Abstract

Stable isotope tracers can be used to quantify the activity of metabolic pathways. Specifically, ^2^H-water is quite versatile, and its incorporation into various products can enable measurements of carbohydrate, lipid, protein and nucleic acid kinetics. However, since there are limits on how much ^2^H-water can be administered and since some metabolic processes may be slow, it is possible that one may be challenged with measuring small changes in isotopic enrichment. We demonstrate an advantage of the isotope fractionation that occurs during gas chromatography, namely, setting tightly bounded integration regions yields a powerful approach for determining isotope ratios. We determined how the degree of isotope fractionation, chromatographic peak width and mass spectrometer dwell time can increase the apparent isotope labeling. Relatively simple changes in the logic surrounding data acquisition and processing can enhance gas chromatography-mass spectrometry measures of low levels of ^2^H-labeling, this is especially useful when asymmetrical peaks are recorded at low signal:background. Although we have largely focused attention on alanine (which is of interest in studies of protein synthesis), it should be possible to extend the concepts to other analytes and/or hardware configurations.

## 1. Introduction

Stable isotopes are commonly used to study metabolic dynamics [1]. For example, rates of biochemical flux can be quantified by measuring the temporal change in enrichment of an isotopically labeled substrate and that of its downstream products. In particular, the administration of ^2^H-water is quite versatile, and applications have been developed to capture the kinetics of carbohydrate, lipid, protein, DNA and RNA [2,3,4,5,6,7]. In many cases it is necessary to detect the incorporation of low levels of ^2^H in a product of interest (e.g., the rate of synthesis may occur at a few percent of the pool per day). In those instances, isotope ratio mass spectrometers (IRMS) are typically used to measure “low enrichments” [8]. For example, IRMS are configured with parallel collecting cups (or detectors), that are set at different resistance to amplify the low ion current that is derived from the less abundant (heavy isotope) signal [8]. In cases where “higher levels” of ^2^H are expected, one can rely on more commonly available mass spectrometers to measure isotope abundance (e.g., quadrupole-based instruments) [9].

When samples are analyzed by coupling a chromatography step to a mass spectrometer (e.g., GC-q-MS), isotope labeling (or enrichment) is measured by comparing the areas of the chromatographic peaks which represent the labeled and unlabeled molecules of a given species; the separate ion chromatograms are processed independent of each other and are compared to their respective baselines [10,11]. We previously reported a novel integration method for determining the isotopic enrichment of known analytes; the unlabeled molecules were used to guide the integration of the labeled molecules, i.e., the separate ion chromatograms (signals) were processed in a dependent manner [12].

Since ^2^H-labeled molecules typically elute earlier than their respective unlabeled forms, it is possible to quantify low levels of ^2^H-enrichment by integrating the leading edge of a chromatographic peak(s) [12]. Biasing the chromatographic integration method increases the measured:expected (M:E) ratio of ^2^H-labeling, and this approach removes data that do not contain information regarding isotope labeling. Although this allowed us to expand the application of ^2^H-water for determining the contribution of gluconeogenesis to glucose production, samples were assayed under conditions that are generally assumed to be ideal, i.e., symmetrical peaks were recorded at high signal:background (S:B) [12]. Herein, we have considered (i) how the degree of isotope fractionation impacts the isotope ratio, (ii) how chromatographic peak width impacts the isotope ratio and (iii) whether uncontrolled variations in the isotope fractionation would influence the isotope ratio. Specifically, we tested whether our approach could facilitate cases in which the analytical conditions are presumed to be less than ideal, i.e., when asymmetrical peaks are recorded at low S:B. In cases such as these it is difficult to reliably measure the entire peak areas, especially the area of the labeled species since it is typically present at a lower abundance than the unlabeled species. The studies reported herein have considered a theoretical examination and direct experimentation.

## 2. Results and Discussion

The ability to measure “small changes” in isotope abundance allows investigators to determine the kinetics of low-turnover species and to better resolve time-dependent changes in metabolism following perturbations. Previous reports have demonstrated clever approaches for coupling GC-q-MS analyses with certain tracer paradigms in order to detect the incorporation of small quantities of an isotope into selected products. For example, one can administer a heavily substituted tracer (e.g., [^2^H_5_]phenylalanine) and readily detect its incorporation into protein since the ^2^H-labeled species is present in a region of the mass spectrum where there is virtually no contribution from the naturally occurring isotopes [13]. In addition, to avoid measuring isotope ratios over a large dynamic range, investigators measure the isotope ratio by comparing the exogenous (administered) isotope against a naturally occurring heavy mass isotopomer, e.g., M5_exogenous phenylalanine_ vs. M3_endogenous phenylalanine_. The application of those novel approaches has proven to be useful in metabolic investigations [13,14].

We, and others, have used ^2^H-water to quantify metabolic rates [2,3,4]. Although ^2^H-water is a versatile tracer, its use in precursor:product investigations typically requires that one quantify the ^2^H-labeling of products in the M1 mass isotopomer. Consequently, investigators are challenged with measuring relatively low levels of ^2^H-labeling over a relatively high background from naturally occurring stable isotopes. Again, strategies have been used to address this type of problem. For example, in favorable cases the background labeling can be reduced by using derivatives in which ^13^C is substituted for ^12^C [15,16]. We enhanced the detection of ^2^H-labeling by capitalizing on the fact that ^2^H-labeled molecules elute slightly earlier than their respective unlabeled species during the chromatographic process [12]. The current study aimed to test paradigms that could extend its utilization.

### 2.1. Simulating and Modeling the Isotope Fractionation

**Figure 1:**
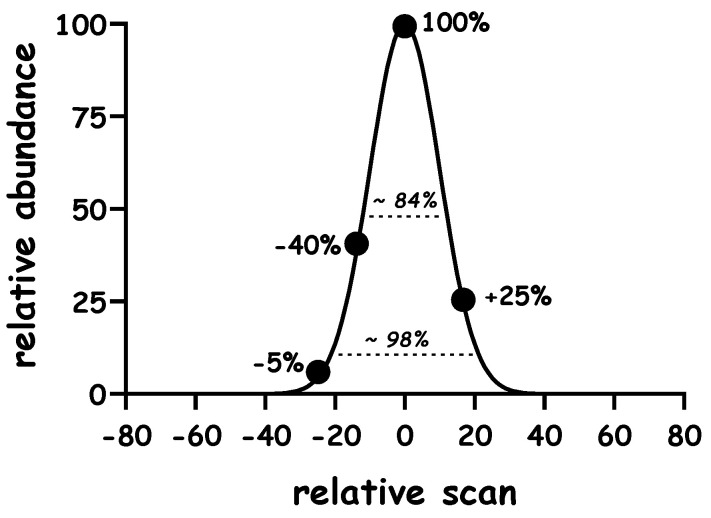
Model chromatographic peak profile. The maximum peak height of the unlabeled analyte (“M0”) to 100%, signals on the leading edge (left) and tailing edge (right) are denoted with a negative and positive sign, respectively, and expressed as a percent of the maximum peak height. Dashed lines drawn at ±1 or 2 σ represent ~84% or ~98% of the peak area, respectively).

Figure 1 outlines general definitions and principles related to these studies, and reference points are included to facilitate the discussion. The notation describing peak height and leading and tailing elution profiles are the same as those used before [12].

**Figure 2:**
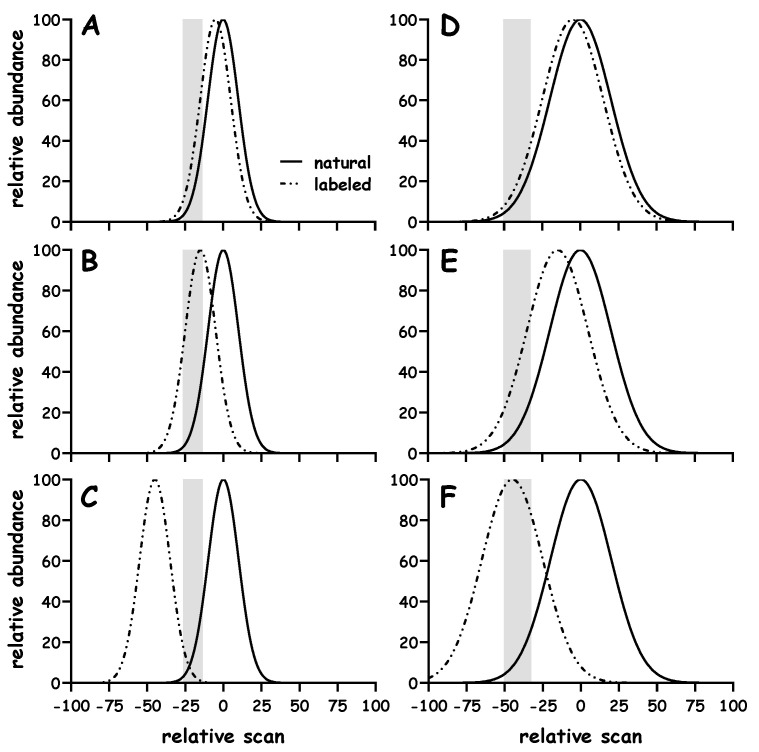
Simulation of isotope fractionation and calculation of measured:expected isotope ratio. Model Gaussian peaks were used to examine how isotope fractionation influences the measured:expected isotope ratio. Simulations considered peaks with a standard deviation of either 10 or 20 scans, Panel (**A**–**C**) or (**D**–**F**), respectively. In each case, the “unlabeled” (natural) peak remained fixed (with its maximum height at scan 0) and the retention of the “labeled” peak was shifted by either −5 (Panel (**A**,**D**)), −15 (Panel (**B**,**E**)) or −45 scans (Panel (**C**,**F**)). Signals were added across the same region in each data set (e.g., ~−1 to ~−20% of the “unlabeled” (natural) peak, shown using the lightly shaded box); in all examples the expected ratio of labeled:unlabeled (natural) signals is 1 but the measured ratio varied from ~2.6, 8.8 and 1.8 in Panel (**A**–**C**) and ~1.7, 3.8 and 12.6 in Panel (**D**–**F**), respectively.

We include a high-level example to orient readers around the studies to be discussed (Figure 2). An unlabeled peak of constant height and width will remain at a set point (i.e., scan 0), a ^2^H-labeled peak of equal height and width elutes earlier (i.e., scan < 0). A fixed area, based on a specific range of scan numbers (defined by the unlabeled peak), is then used to quantify the respective signal intensities of both peaks and determine the isotope ratio. Various outcomes can be expected depending on the degree of fractionation. For example, the lesser the separation the closer the M:E ratio is to the true value of 1 (Figure 2A vs. Figure 2B and Figure 2D vs. Figure 2E). Although there is an opportunity to increase the M:E isotope ratio in cases where fractionation is enhanced (e.g., Figure 2A vs. Figure 2B), this effect is lost if fractionation is increased beyond certain limits (e.g., Figure 2B vs. Figure 2C). As one can infer from Figure 2, the ability to increase the M:E isotope ratio depends on the degree of isotope fractionation and the ranges over which the data are integrated. Our simulation and modeling studies determined how certain factors would influence the M:E isotope ratio, specifically, the effect of (i) peak width and integration region (including sampling density), (ii) S:B and (iii) error in the fractionation.

Other simulation and modeling studies further examined the concepts outlined in Figure 2. As the range becomes smaller one can observe a greater increase in the M:E isotope ratio. For example, comparing the range −1% → −20% vs. −1% → −5% vs. single point at –1%, in Figure 2B, results in ratios of 8.8 vs. 16.7 vs. 29.2, respectively.

A second area where simulation and modeling studies provided insight concerns the peak width. As discussed above, studies considered the fractionation of two peaks of equal height and area, e.g., Figure 2A–C vs. Figure 2D–F contrasts the effect of a standard deviation (σ) of 10 vs. 20, respectively. Although comparable increases can be observed in the M:E isotope ratio, when integrating over the leading edge (i.e., −1% → −20%, marked with the shaded box), the broader peaks allow more flexibility (or buffering) in cases where integration ranges are modified. Wider peaks can limit some of the sharp dropouts in the M:E isotope ratio that can result with narrow peaks (e.g., Figure 2C vs. Figure 2F).

### 2.2. Using GC-MS Analyses to Test the Model Predictions

**Figure 3:**
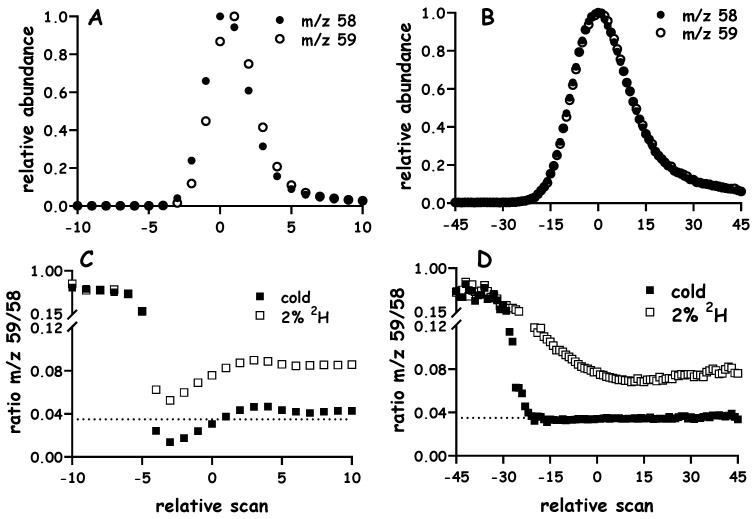
Effect of dwell time on chromatographic resolution of isotope ratios. Samples of natural abundance acetone were analyzed using GC-q-MS to demonstrate the effect of dwell time; selected ion monitoring was performed on *m/z* 58 and 59 at 100 or 10 ms per ion (Panel (**A**) or (**B**), respectively); the respective 58 and 59 signals were normalized against the highest intensity for a given *m/z* and overlaid to better visualize the data, scan 0 represents the maximum signal for *m/z* 58. The ratio of *m/z* 59 to 58 was plotted at each scan, a dotted line is included to represent the theoretical background value (i.e., ~0.035) that is expected (Panel (**C**) contains 100 ms dwell time and Panel (**D**) contains 10 ms dwell time). Panel (**C**,**D**) also include a representative plot from an acetone standard made via incubation in 2% enriched ^2^H-water.

It is important to note that peak width (and scans across a peak) can be influenced by (i) the chromatographic conditions, i.e., the column type, temperature gradient and derivative, and (ii) the mass spectrometry acquisition parameters, e.g., the dwell time for selected ion monitoring. We refer to this later point as “sampling density” and develop a discussion of its merits using data obtained via the analyses of acetone (Figure 3).

It is of interest to note that when ^2^H-water is used to study biochemical flux, the ^2^H-labeling of acetone acts as a proxy for precursor exposure [3,17]. Figure 3 demonstrates the raw signals that are acquired when naturally labeled acetone is analyzed using GC-q-MS, spectra were collected using EI and SIM of *m/z* 58 and 59 at a dwell time of 100 or 10 ms per ion (Figure 3A or B, respectively). The respective signals correspond to the M0 and M1 mass isotopomers of acetone, data were normalized against the greatest signal for a given ion in a run and plotted, and this helps to better visualize the profiles; note that *m/z* 59 is expected to be ~3.5% that of *m/z* 58, which would be difficult to see if data were not normalized.

Although a longer dwell time (e.g., 100 ms vs. 10 ms) leads to a reasonable number of data points on which to estimate the peak area (Figure 3A vs. Figure 3B, respectively), a plot of the 59/58 ratio in each demonstrates the utility of reducing the dwell time and increasing the data acquisition. The expected ratio of 59/58 is ~0.035 (dotted line in Figure 3C,D). In cases where a short dwell time (e.g., 10 ms) is used, it is possible to correctly estimate the ratio using a single (or a few) points around the peak maximum, in contrast, collecting data using longer dwell times (e.g., 100 ms) does not lead to a stable ratio as the peaks elute and, therefore, obligate one to integrate the entire (or majority) of the peaks (note the solid squares in Figure 3C vs. Figure 3D, respectively). Figure 3 also contains an example of the 59/58 ratio in cases where a sample of ^2^H-enriched acetone was analyzed. Although the 100 ms dwell time was able to differentiate the labeling from natural acetone using virtually any scan(s) (Figure 3C, open squares), when data are collected using 10 ms dwell times (Figure 3D, open squares) there is a marked bias at the earlier scans (e.g., ~−20 to ~0 scans shows a greater difference from the natural acetone). Capitalizing on this bias was the subject of deeper investigation using alanine as a model analyte.

**Figure 4:**
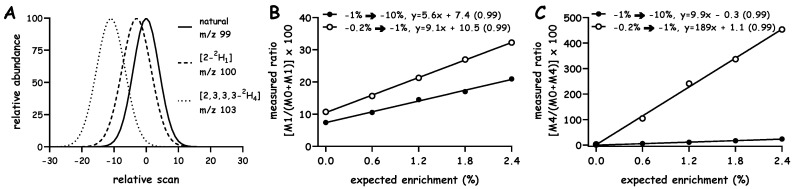
Experimental use of isotope fractionation to amplify the measured:expected isotope ratio. Mixtures containing equal quantities of naturally labeled (solid line), [2-^2^H_1_]labeled (dashed line) and [2,3,3,3-^2^H_4_]labeled (dotted line) alanine were converted to their “methyl-8” derivatives and analyzed under electron impact ionization using selected ion monitoring of *m/z* 99, 100 and 103 (10 ms dwell per ion) (Panel (**A**)). Mixtures containing unlabeled and either [2-^2^H_1_]alanine (Panel (**B**)) or [2,3,3,3-^2^H_4_]alanine (Panel (**C**)) were analyzed and signals were integrated over various regions of the ion chromatograms; the region over the which the integration was run and the resulting linear fits are shown in each panel (r^2^ in parenthesis, *n* = 3 replicates, data shown as mean).

Readers will likely recognize that increasing the number of data points across the peak improves the line shape to some degree, however, there should be a balance between a rapid (low sensitivity) and slower (high sensitivity) acquisition. In fact, one expects that a longer dwell time will result in better ion statistics since more time will be spent collecting a signal, however, sitting on one ion will immediately limit the collection of signal from another ion [10]. In our case, we aim to measure the ratio of (at least) two ions; therefore, we were concerned that longer dwell times effectively lead us to miss signal as the analytes elute and that the isotope fractionation that occurs during chromatography will be missed. We can appreciate that dwell times which are too short can lead to noise in the data too; we have seen this in some circumstances and presumably reflects electronic noise that comes from rapid switching between *m/z* channels. Although we have typically observed reproducible data with the current setting of 10 ms per ion, investigators should not take this as a hard or fixed value, investigators should consider the dwell time for their specific application. As expected, [2,3,3,3-^2^H_4_]alanine elutes before [2-^2^H_1_]alanine which elutes before naturally enriched alanine (Figure 4A). Studies analyzed mixtures containing known quantities of the respective alanine standards (Figure 4B,C, respectively). Samples were integrated across different ranges and linear responses in the M:E isotope ratio were observed. These data support predictions from the simulation and modeling studies (Figure 2) and demonstrate points of new knowledge regarding the performance of GC-q-MS analyses.

First, the ability to amplify the signal is sensitive to the region over which the integration is performed. For example, progressively decreasing the integration region leads to an increase in the response factor (Figure 4B). Second, much greater amplification can be achieved in the case when there is more isotope fractionation. For example, when analyzing [2-^2^H_1_]alanine we could achieve ~9-fold amplification whereas when analyzing [2,3,3,3-^2^H_4_]alanine we could achieve ~189-fold amplification (Figure 4B vs. Figure 4C, respectively). Third, it was possible to reliably quantify the amount of isotope present in each sample using even a single data point (not shown). These observations are consistent with the predictions from simulation and modeling studies (Figure 2) and agree with our earlier study [12]. The fact that linear responses were observed when even a single data point was used to construct the calibration plots (not shown) suggests that isotope fractionation is highly consistent over multiple injections. Although we are intrigued by the fact that highly reproducible data can be obtained with such limited sampling, it is presumably better to utilize several data points when measuring the isotope labeling. Readers are referred to excellent discussions regarding the impact of hardware configuration and ion statistics on the measured isotope ratio [10,11,18].

### 2.3. Enhancing Isotope Ratio Analyses in Cases of Poor Chromatography and/or Low Signal Intensity

Our previous studies relied on conditions that are generally assumed to be ideal, S:B was reasonably high and chromatographic peaks were symmetrical [12]. Herein, we tested whether the logic would facilitate analyses that are run under conditions which might be considered unsuitable. For example, the studies noted above demonstrate that isotope fractionation is robust, and our logic effectively collects the “best” data signals (in a manner which is consistent across different samples) (Figure 4). Since our approach allows us to selectively collect data in virtually any region of the chromatogram, we hypothesized that this might be especially useful when signals deteriorate and/or when elution profiles are not robustly defined.

**Figure 5:**
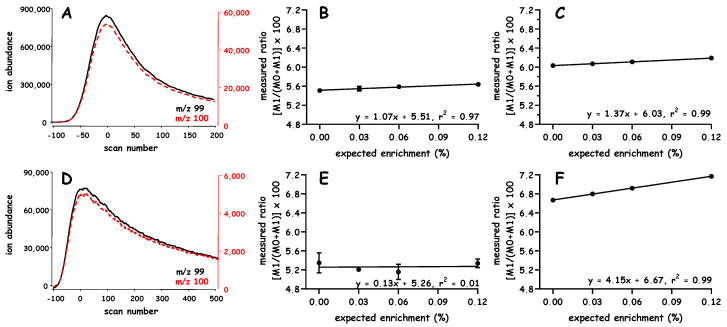
Influence of chromatographic symmetry and signal:background on the integration of alanine standards. Standards containing known mixtures of alanine and [2-^2^H_1_]alanine were analyzed using the “methyl-8” derivative and GC-q-MS. The ion chromatograms of *m/z* 99 and 100 (Panel (**A**), black and red, respectively) characterized by reasonably high signal:background and modest asymmetry (the peak width at half-height is −40 scans and +60 scans, the x-axis set the maximum intensity for *m/z* 99 to scan 0). Panel (**A**) contains an example of naturally labeled alanine. Panel (**B**) demonstrates the results when using the commercially available software to measure the respective peak areas. Panel (**C**) demonstrates the result that is obtained when our manual integration method is used (integration was performed from −1.0% → +25% of the peak). Panel (**D**) contains an example of the same standards but run under conditions of ~10-fold lower intensity and more marked asymmetry (the peak width at the half-height is −50 scans and +200 scans, again, the x-axis set the maximum peak height of *m/z* 99 at scan 0). Panel (**E**) demonstrates the results that are obtained when using the commercially available software to measure the peak area (similar data were obtained using peak height, not shown). Panel (**F**) demonstrates the result that is obtained when using our manual integration method, integration was performed from −5% → −25% of the peak (*n* = 4 replicates of each standard, data are shown as mean ± sem).

We first acquired data using a set of standards containing known mixtures of alanine and [2-^2^H_1_]alanine, and there was relatively high S:B (maximal height of *m/z* 100 at scan 0 is ~6000 times its baseline intensity) but noticeable asymmetry (the width at the half-height is approximately −40 scans and +60 scans) (Figure 5A). We compared the ratio of *m/z* 100 to 99 using “AutoIntegrate” outputs of the total area against an integration of a limited region of the leading edge (note that “AutoIntegrate” also yielded a measure of peak height, in this case peak area and peak height generated comparable isotope ratios, not shown). The linear response in the M:E isotope ratio of the standards demonstrated that one could detect the ^2^H-labeling; however, the marked variation with the “AutoIntegrate” routine made it difficult to resolve small changes between these standards (Figure 5B). In contrast, our partial integration method reduced the variation between the measurements of a given standard (Figure 5B vs. Figure 5C) and allowed us to differentiate between samples containing background ^2^H-labeling and those containing low levels of [2-^2^H_1_]alanine (*p* < 0.01, Figure 5C). Although highly reproducible isotope ratios were observed when using our integration, the lower response factor (i.e., the slope of the regression analysis was ~1.4) restricted the detection to ~0.03% ^2^H above the natural background.

Further reflection led us to test a case where there was lower S:B (maximal height of *m/z* 100 at scan 0 is ~50 times its baseline) and more marked asymmetry (the width at the half-height is approximately −50 scans and +200 scans) (Figure 5D). We reanalyzed the same standards described above and again tested the different data integration routines. Using the “AutoIntegrate” routine it was not possible to reliably quantify the incremental increase in excess ^2^H in any of the standards (Figure 5E). However, using our method to perform a partial integration of the peaks demonstrated that it was possible to detect ~0.03% excess ^2^H_1_ over natural background, i.e., measuring the isotope ratios between −5 and −25% of the peak maximum leads to a 4-fold increase in the M:E isotope ratio without compromising the precision of the measurements (Figure 5F).

**Figure 6:**
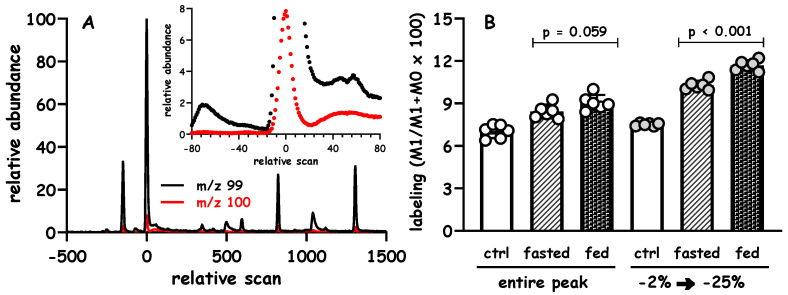
Practical utility for limiting integration boundaries during GCMS analyses. Rats were given ^2^H-water and randomized to fasted or fed groups, the incorporation of ^2^H-alanine into total liver proteins was determined (*n* = 6 per group). Panel (**A**) demonstrates a typical chromatogram that is observed using the “methyl-8” derivative and SIM of 99 and 100 (10 ms per ion dwell time); the inset expands the region surrounding alanine. Panel (**B**) demonstrates the ^2^H-labeling of protein-bound alanine following hydrolysis, the 99 and 100 signals were integrated using standard parameters from the vendor software (to quantify the entire peak) or using a limited region (from −2 to −25% of the peak).

Figure 6 demonstrates the practical utility of this approach when rat liver protein is hydrolyzed and analyzed using SIM. The time scale was normalized to set alanine at a relative scan of 0, the inset expands the region of interest surrounding the 99 and 100 signals (Figure 6A). Using these conditions, protein synthesis was measured in fasted vs. fed rats following the administration of a bolus of ^2^H-water. Regardless of the integration method, we could detect the presence of ^2^H-alanine in animals given ^2^H-water vs. naïve controls (i.e., “ctrl”, not given ^2^H-water). Although one can visualize an expected apparent stimulation of protein labeling in fed vs. fasted rats in cases where the entire peak is integrated, the data become tighter when integrations are restricted to a smaller boundary (Figure 6B). In order to determine the true enrichment, these samples would need to be corrected using a set of known standards that are analyzed in parallel and use the same integration boundaries. 

### 2.4. Additional Considerations of the Theory

In total, our observations strongly support a rationale for customizing integration routines; a novel advantage is gained when it is of interest to quantify ^2^H-labeling since isotope fractionation can facilitate studies. Although the experimental data agree with the simulation and modeling studies, we identified two final questions to address.

First, does the positional location of ^2^H influence the isotope fractionation? For example, in studies where ^2^H-water is administered, a single ^2^H atom can be substituted for any one of several carbon-bound hydrogens in a product. To test whether the positional location of ^2^H impacts the degree of isotope fractionation we generated positional isotopomers of [^2^H_2_]sorbitol; [6,6-^2^H]glucose was reduced with NaBH_4_ and [5-^2^H]glucose with NaB^2^H_4_ to generate [6,6-^2^H_2_] and [1,5-^2^H_2_]sorbitol, respectively. We also examined positional isotopomers of [^2^H_1_]palmitate, by reducing 2-bromopalmitate and 16-bromopalmitate with NaB^2^H_4_ we were able to generate [2-^2^H_1_] and [16-^2^H_1_]palmitate, respectively. Each labeled species was mixed with an equal amount of its respective unlabeled species and analyzed by GC-MS (sorbitol as hexaacetate and palmitate as methyl-ester derivatives). Using this approach, we did not detect any influence of positional labeling on the degree of isotope fractionation (not shown); therefore, it appears that the number of isotopic substitutions and not the location of ^2^H influences isotope fractionation.

Second, we tested whether we could modulate the fractionation of singly-labeled alanine. As noted earlier, we often administer ^2^H-water and measure the labeling of product molecules in the M+1 species ^2^. It appears that for the assay of alanine reported here, we are at/near the limit of resolution. Namely, we tested the “methyl-8” derivative on a 30 m vs. a 50 m DB17ms column (at the same temperature programs described in Materials and Methods), but we could not observe a substantial effect on fractionation of unlabeled and [2-^2^H_1_]alanine. Presumably this is expected since the oven temperature gradient is already slow, namely, increasing at 5 °C per minute. Subsequent tests compared the fractionation of the unlabeled and [2-^2^H_1_]alanine on 30 m column with a more polar phase (i.e., a OV225, at the same temperature gradient as the 30 DB17ms column). That test resulted in substantially less fractionation (~3 vs. ~7 scans using the OV225 vs. the DB17ms, not shown), suggesting that less polar columns and/or derivatives may increase the fractionation.

## 3. Materials and Methods

### 3.1. Chemicals and Supplies

Unless specified, all chemicals and reagents were purchased from Sigma-Aldrich (St. Louis, MO, USA). ^2^H-water (99.9 atom percent excess), [2-^2^H_1_]alanine (98.9 atom percent excess) and [2,3,3,3-^2^H_4_]alanine (98 atom percent excess) were purchased from Cambridge Isotopes (Andover, MA, USA). GC-MS supplies were purchased from Agilent (Wilmington, DE, USA). Standards containing unlabeled and labeled molecules were prepared by mixing known quantities of the respective compounds.

### 3.2. Biological

Metabolic labeling was achieved in fasted vs. fed Wistar Han rats by administering a bolus of ^2^H-water (20 uL per g body wt) at ~3 PM animals were then randomized to subgroups that were either allowed free access to food overnight or maintained in cages with no food overnight. The following morning (~9 AM) all rats were euthanized, and blood and tissue were collected. The ^2^H-labeling in total liver protein was determined following homogenization in 10% TCA. Protein pellets were transferred to a new tube and spun at 10,000 rpm at 4 °C for 10 min, samples were washed two times with 10% TCA to remove all free amino acids and dried under a stream of warm nitrogen. Samples were then resuspended in 250 uL 6 N HCl and heated at 85 °C overnight. A 25 uL aliquot of the protein hydrolysate was transferred to a new tube and dried under a stream of heated nitrogen. GC-MS analyses were performed as described below using the “methyl-8” derivative. Studies were approved by the Merck Research Laboratories Institutional Animal Care and Use Committee (#2020-601166-Aug); note that these animals are controls not published previously [2].

In other studies, metabolic labeling was achieved by feeding 12 week old male mice a diet containing 99% [5,5,5-^2^H_3_]leucine (Mouse Express L-LEUCINE, Cambridge Isotopes; Andover, MA). The mice ate ad libitum. Comparable studies administered ^2^H-water to 12 week old mice (intraperitoneal injection, 20 μL per g of body weight) to enrich the body water to ~2.5% ^2^H. Mice were returned to their cage and 5% ^2^H-water was added to their drinking water, mice drank ad libitum to maintain the enrichment at ~2.5% ^2^H in total body water. After 14 and 10 days, respectively, the mice were anesthetized with isoflurane and euthanized by cervical dislocation and exsanguination. Hearts were excised, flash frozen in liquid nitrogen, and stored at −80 °C. Studies were approved by the Institutional Animal Care and Use Committee at University of Vermont (#19-032) and were in accordance with the guidelines listed in the Guide for the Use and Care of Laboratory Animals published by the National Institutes of Health. The review criteria are consistent with the “ARRIVE guidelines” that are endorsed by Metabolites. 

### 3.3. Analytical

The ^2^H-labeling of water was determined after exchange with acetone. Briefly, assays were run in V-shaped 96-well plates by adding 5 μL of sample, 4 μL 10 N NaOH and 3 μL of acetone. Wells were immediately capped with septa, and plates were then spun to collect all liquid at the bottom of the well and left at room temperature for 4 h. Headspace analyses were performed using an Agilent 5973N-MSD equipped with an Agilent 6890 GC system (Santa Clara, CA, USA), a DB17-MS capillary column (30 m × 0.25 mm × 0.25 µm; temperature held at 175 °C for 1.75 min, helium flow 1 mL per min, split ratio 20:1, inlet and transfer line temperatures were held at 250 °C and 290 °C, respectively), data were acquired using selected ion monitoring (SIM) under electron impact ionization (EI), mass-to-charge ratio (*m/z*) 58 and 59 (dwell time = 10 ms per ion) [17]. Note that *m/z* 58 and 59 represent ions from intact acetone and its M1 isotope, respectively.

The ^2^H-labeling of alanine was determined as follows. The “methyl-8” derivative was formed by reacting alanine with acetonitrile, methanol, and “methyl-8 reagent” (*N*,*N*-dimethylformamide dimethyl acetal [19]; Pierce, Rockford, IL) reagents were mixed at a volume ratio of 1:2:3, 100 μL was added to a dry sample which was then capped and heated at 75 °C for 30 min. Following this derivatization step, samples were analyzed using an Agilent 5973N-MSD equipped with an Agilent 6890 GC system (Santa Clara, CA, USA), a DB17-MS capillary column (30 m × 0.25 mm × 0.25 µm). The temperature program was: 90 °C initial, hold for 5 min, increase by 5 °C per min to 130 °C, increase by 40 °C per min to 240 °C and hold for 5 min (inlet and transfer line temperatures were held at 250 °C and 290 °C, respectively), alanine elutes at ~12 min. The split ratio was varied between 5:1 and 50:1 for different samples according to the desired conditions, with a helium flow of 1 mL per min. The mass spectrometer was operated in the EI mode which yields a molecular ion at *m/z* 158 (containing all of the carbon-bound hydrogens), a fragment ion at *m/z* 143 (containing only the hydrogen bound to the α-carbon) and a fragment ion at *m/z* 99 (containing all four carbon-bound hydrogens of alanine). We measured the enrichment of alanine using SIM of the fragment at m/z 99 for naturally labeled alanine and 100 and 103 for [2-^2^H_1_] and [2,3,3,3-^2^H_4_]alanine, respectively, with a dwell time = 10 ms per ion. Note that the fragment at *m/z* 99 represents the base ion in the spectrum, whereas the molecular ion (*m/z* 158) accounts for ~15% of the intensity of the base ion. In studies where ^2^H-water is used to quantify protein synthesis, this derivative and fragment provide an advantage since the background isotope abundance has limited influence by any extraneous signal, and the use of DB-17ms column facilitates a good separation between alanine and glycine [20].

Tryptic peptides derived from heart proteins were generated and analyzed as follows. Individual pieces of intact heart muscle (2–4 mg), were solubilized in RapiGest SF Surfactant (Waters Corporation), reduced, alkylated and digested with trypsin (Promega) as described [21]. The resultant peptides were separated and analyzed via electrospray by coupling ultra-high pressure liquid chromatography (LC, Dionex UltiMate 3000) with a Q Exactive Hybrid Quadrupole-Orbitrap mass spectrometer (Thermo; Bremen, Germany) [21]. Peptides were identified from the mass spectra (MS) using Proteome Discoverer 2.2 (Thermo; Bremen, Germany) and searched against the mouse proteome (downloaded from UniProt). LC elution profiles were generated for mass isotopomers of the DLEEATLQHEATAAALR peptide shared between the myosin isoforms MYH6 and MYH7.

### 3.4. Simulation and Mathematical Modeling of Isotope Fractionation

We assumed that a Gaussian profile would represent the chromatographic data, therefore, Equation (1) was used to construct model peaks:

(1) $$y=\left(\frac{area}{\sigma \sqrt{2\pi }}\right)\;\times {\;}^{e^{-{\left(x-a\right)}^{2}/2\sigma^{2}}}$$

where “*σ*” represents the standard deviation and “*a*” represents the mean (which was set to 0), the peak area was set to 1,000,000 (Figure 1) [22]. The notation used here is the same as that used previously [12], the peak maximum (at scan 0) is set to 100%, signals on the leading edge (left side of the maximum) are described using a “−“ sign and those on the tailing edge (right side the maximum) are described using a “+” sign, *σ* was set to 10 or 20 scans in order to simulate narrow or broad peaks, respectively.

The effect of isotope fractionation on the amplification of the M:E isotope ratio was simulated using two assumptions. First, we assume that the model peaks represent the elution profiles of the labeled and unlabeled molecules. Second, for simplicity, we assume an equal proportion of labeled and unlabeled molecules, i.e., in all cases the expected ratio of the peaks is 1. Simulations were performed under conditions where the unlabeled peak remained fixed and the labeled peak was shifted by −5, −15 or −45 scan units on the x-axis (analogous to the effect of increasing isotope fractionation during chromatographic elution). The ratio of the peaks was then determined over a small region of the leading edge of the fixed peak, e.g., when the signal representing the fixed peak was ~−1 to ~−20% of its maximum (e.g., noted by a shaded box in Figure 2). Signals were added for the respective fixed and fractionated peaks, and the ratio of these sums was calculated.

### 3.5. Processing GC-MS Data Following the Analyses of Alanine Standards

Chromatographic integration: “Auto-Integrate” method.

Ion chromatograms were quantified using the “Auto-Integrate” function that is available with the Agilent Chemstation software. Default integration parameters were used in all assays including, initial area reject = 1, initial peak width = 0.02 min, shoulder detection = OFF and initial threshold = 18. The SIM window remained open for ~0.5 min on either side of the peaks. The peak area and peak height for *m/z* 99 and 100 were recorded and used to calculate the ^2^H-labeling.

Chromatographic integration: Manual method.

The data were exported using the Agilent Chemstation feature “Export 3-D data”, unless specified, the range was set to export the necessary ions for alanine (e.g., *m/z* 99, 100 and 103 for M0, M1 and M4, respectively) covering ~500 scans of baseline before the peak elutes and ~800 scans after the maximum peak height. The exact range is not critical, one should include enough data points to describe the peaks; output files are in the form “.csv”.

Before proceeding with the integration, all chromatograms were manually inspected to determine whether the abundance of alanine was similar (M0 signals did not vary by more than ~3-fold across samples). Under good analytical conditions (i.e., when symmetrical peaks were observed and there was high S:B), the maximum peak heights of *m/z* 99 and *m/z* 100 were typically >300 times the background trace. The raw data (scan number and ion abundance of *m/z* 99 and *m/z* 100) were then imported to a MS Excel spreadsheet.

## 4. Conclusions

In summary, the isotope fractionation that occurs during gas chromatography can be used to enhance the detection of ^2^H-labeling. Since the fractionation that occurs during gas chromatography is highly reproducible and apparently not affected by the positional location of ^2^H substitutions, our studies suggest that strategies which optimize the degree of isotope fractionation should provide a useful means of increasing the detection of ^2^H-labeling when samples are analyzed using more conventional mass spectrometers. Our logic may help circumvent a reliance on more traditional IRMS in certain cases. 

We should note that a dependency on matrix purity and peak shape is not necessarily critical (impurities can influence our biased integration just as they can influence conventional approaches for integrating peaks), the labeled and unlabeled molecules simply need to behave in a comparable fashion. We should also emphasize that although the absolute retention of an analyte of interest will certainly vary across injections (e.g., >200 ms for the analyses of alanine), the fractionation of labeled and unlabeled molecules shows virtually no variation under the conditions applied herein (e.g., <10–20 ms). As implied, the stability of isotope fractionation is imperative for guiding peak integrations. Although we found that one can rely on a single dwell cycle to determine the ratio of unlabeled to labeled molecules (e.g., 10 ms for each ion), we do not advocate using such an extreme slice of the data; nevertheless, our observations make the case that isotope fractionation is remarkably consistent. In addition, we expect that the ability to detect ions at greater frequency will also improve the use of isotope fractionation as a means for amplifying the apparent ^2^H-labeling. For example, one can use smaller regions of the leading edge of the peak while still including a reasonable number of data points in the calculation. Lastly, when using our biased integration logic, it is imperative to include known standards if the goal is to correct the data and obtain true enrichments; one can obtain relative differences between groups without the use of known standards (e.g., Figure 6B). 

Based on the experimental data presented here, it appears that there is a need to develop alternative software to process data obtained during GC-MS assays. We should note that our studies are not meant to suggest that GCMS analyses can be a substitute for IRMS analyses. Although our data suggest that one can blur the lines between the analytical methods, a head-to-head comparison would be necessary to identify true limits. We should also add that advances in high-resolution mass spectrometry can offer novel advantages in cases where one aims to quantify low levels of labeling [23], especially if multiple stable isotopes have been administered [24].

**Figure 7:**
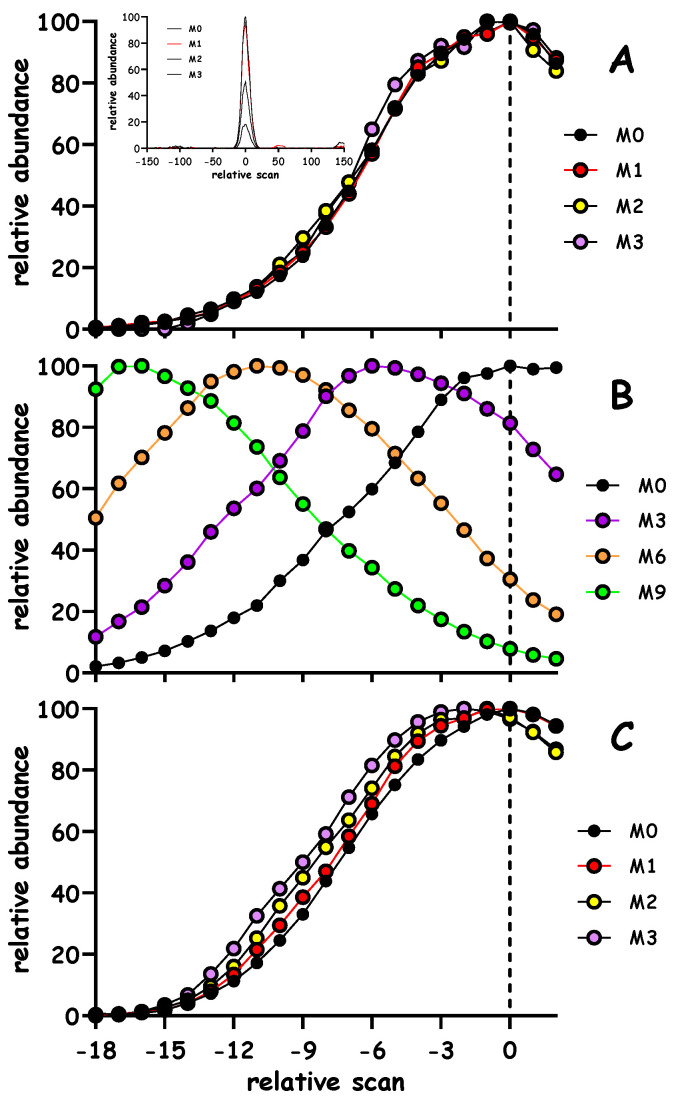
LC-based separation of ^2^H-labeled peptides. Mice were either fed a diet containing 99% [5,5,5-^2^H_3_]leucine or given ~2.5% ^2^H-water for ~2 weeks, cardiac proteins were extracted, digested with trypsin and subjected to LC-MS full scan analyses. Panel (**A**) contains an example of the elution profiles of the M0→M3 mass isotopmers for DLEEATLQHEATAAALR^2+^ (*m/z* 919.97 for M0), each mass isotopomer was normalized to its respective maximum peak height; the plots overlay each other. The inset of Panel A contains a wider profile of the LC peak which elutes at ~44 min, as demonstrated, the extracted chromatograms for *m/z* 919.97 and the respective isotopes are relatively free of any contamination. Panels (**B** and **C**) contain analogous plots in cases where ^2^H_3_-leucine or ^2^H-water are given, respectively. As exemplified in Panel B, the incorporation of more leucines will lead to marked shifts in the elution profiles which are readily visible since the precursor was so heavily labeled. Panel C demonstrates a shift in the elution profile of M1→M3 species when ^2^H-water is used as a tracer, albeit a more subtle shift is expected since body water was only enriched ~2.5% in ^2^H.

Our simulation and modeling studies support the notion that partial integrations would be applicable to other analytical formats and instrument configurations e.g., liquid chromatography-mass spectrometry. In fact, Figure 7 outlines an example where tryptic peptides were analyzed using full scan mass analyses of heart DLEEATLQHEATAAALR, a peptide shared between myosin isoforms MYH6 and MYH7. To better visualize the chromatographic separation of the ^2^H species, each mass isotopomer was normalized to its maximum peak height. As expected, in a control animal (i.e., when no ^2^H tracer is administered) the mass isotopomers are superimposed on each other (Panel A). However, the administration of [^2^H_3_]leucine or ^2^H-water results in marked shifts of the labeled species (Panel B and C, respectively). We expect that one should be able to capitalize on this isotope fractionation in cases where ^2^H-labeled tracers are administered.

We should note that attention towards integration strategies is not limited to studies involving fractionation, this has merit for various chromatographic applications [11,18].

Finally, we have not commented on how data are used in different metabolic flux models; indeed, this is very context-dependent. Fortunately, there is strong interest in developing tools for supporting related areas of tracer-based research [25,26,27].
